# The Information Technology Infrastructure for the Translational Genomics Core and the Partners Biobank at Partners Personalized Medicine

**DOI:** 10.3390/jpm6010006

**Published:** 2016-01-21

**Authors:** Natalie Boutin, Ana Holzbach, Lisa Mahanta, Jackie Aldama, Xander Cerretani, Kevin Embree, Irene Leon, Neeta Rathi, Matilde Vickers

**Affiliations:** Partners HealthCare, 65 Landsdowne Street, Cambridge, MA 02155, USA; aholzbach@partners.org (A.H.); lmahanta@partners.org (L.M.); jAldama@partners.org (J.A.); xCerretani@partners.org (X.C.); kembree1@partners.org (K.E.); iLeon@partners.org (I.L.); nRathi@partners.org (N.R.); mvickers@partners.org (M.V.)

**Keywords:** biobank IT, personalized medicine IT, precision medicine IT, biobank software, biobank information technology

## Abstract

The Biobank and Translational Genomics core at Partners Personalized Medicine requires robust software and hardware. This Information Technology (IT) infrastructure enables the storage and transfer of large amounts of data, drives efficiencies in the laboratory, maintains data integrity from the time of consent to the time that genomic data is distributed for research, and enables the management of complex genetic data. Here, we describe the functional components of the research IT infrastructure at Partners Personalized Medicine and how they integrate with existing clinical and research systems, review some of the ways in which this IT infrastructure maintains data integrity and security, and discuss some of the challenges inherent to building and maintaining such infrastructure.

## 1. Introduction

The software and hardware at the heart of Partners Personalized Medicine’s (PPM) research operations enables the management of large amounts of data, maintains the privacy and confidentiality of subjects participating in research, drives efficiencies in the laboratory, and enables the management of genomic data. Robust and scalable information technology infrastructure that is integrated with clinical systems is a critical component of running the Partners Biobank and Translational Genomics Core at Partners Personalized Medicine, whether the solutions are custom or commercial applications. Key functionality includes managing communications with hundreds of thousands of patients, distributing data from multiple clinical systems, tracking inventory of millions of samples, creating laboratory efficiency by managing/tracking laboratory workflows, and analyzing genomic data from tens of thousands of patients. Information technology is essential to handle the scale of the data that underlies personalized medicine, with the expectation of ensuring data integrity through all pieces of required functionality.

The mission of PPM is to utilize genetics and genomics to improve the care of patients through the promotion and implementation of personalized medicine in caring for patients throughout the Partners HealthCare system and in healthcare nationally and globally. Partners Personalized Medicine combines the Laboratory for Molecular Medicine, a CLIA-certified clinical diagnostics laboratory, the Translational Genomics Core, a research core that performs sequencing, genotyping, and gene expression, and the Partners HealthCare Biobank, a large repository of patient data and samples. The IT infrastuture at PPM supports these three separate but integrated components. In this paper, we discuss and describe the IT infrastructure that supports the Translational Genomics Core and the Biobank.

## 2. Functional Components and Integrations

**Figure 1:**
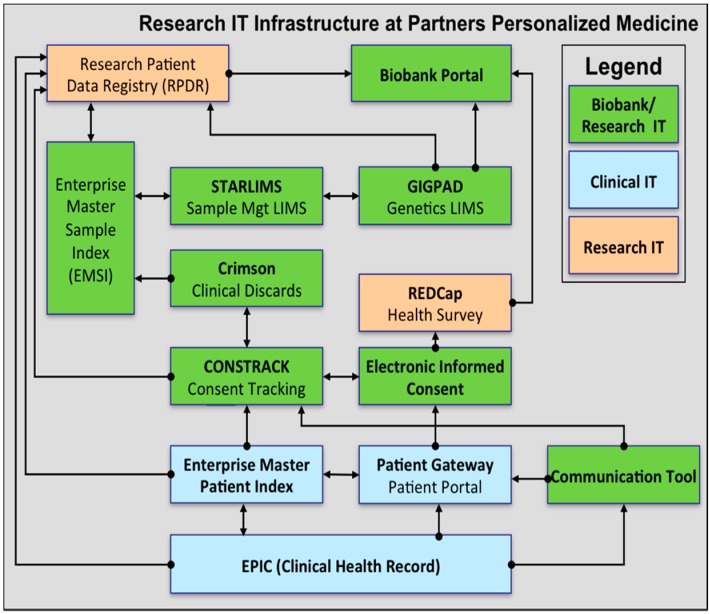
Logical components of the Biobank and Genomics Core IT infrastructure.

Four functional components are at the core of the information technology infrastructure for the Translational Genomics Core and the Partners Biobank: (1) consent tracking, (2) sample management, (3) laboratory processing, and (4) distribution of data and samples. At the Partners Biobank and Partners Translational Genomics Core, these four functional areas are served by several distinct software applications that are integrated with each other (see Figure 1). These biobanking and genomic software applications are further integrated with the clinical infrastructure at Partners HealthCare. The resulting IT infrastructure enables the management of data associated with hundreds of thousands of patients, millions of samples, and clinical data that spans the entire healthcare system with minimal data integrity issues.

The informed consent process is a critical component of biobanking. At Partners HealthCare, an academic medical center that serves 6 million patients across multiple hospitals, a custom application called Constrack manages patient outreach and consent. Constrack integrates with the Enterprise Master Patient Index system at Partners to uniquely identify each patient based on a combination of identifiers including medical record number, name, date of birth, and address. Research Coordinators may log into Constrack to track whether a patient provided consent, refused consent, withdrew consent, was contacted, or is undecided. Some additional statuses that may be selected are “ineligible” and “deferred by MD”, to indicate that the patient should not be approached for consent at that point in time. Constrack manages multiple consent forms for multiple studies as the Biobank collaborates with a number of research studies throughout the hospital system.

A benefit of Constrack being a custom application is the ease and speed with which new functionality can be implemented. In 2014, two large pieces of functionality were launched within a few weeks of conception. The first of these two pieces of functionality, paperless consent, enables research coordinators and subjects to sign the consent form during the in-person consent process using a stylus or their finger on an iPad. The signature is embedded into a pdf of the consent form and stored on a secure server within our institutional firewall. A second enhancement that took just a few weeks to roll out was anintegration with the Enterprise Master Specimen Index (EMSI) that enables research staff to quickly check on the availability of samples for a patient. This new piece of functionality has enabled research staff to better manage sample collection in the many clinics where the Biobank is recruiting patients. Constrack also indicates whether a patient was contacted via email for electronic consent or via post for in-person consent.

In 2014, a new user interface and application layer were built for Constrack that integrates with the Patient Portal at Partners HealthCare, called Patient Gateway. This website enables electronic informed consent (eIC), which has proven to be a successful recruitment strategy for the Biobank. Between June 2014, when it went live, and the end of August 2015, 6603 patients have provided their consent via eIC. A key component for the success of eIC at Partners HealthCare has been the existence of clinical infrastructure to authenticate patients with a unique user name and password.

Once the specimens are collected from consented patients, they are managed in a commercial Laboratory Information Management System (LIMS) called STARLIMS. STARLIMS manages sample inventory as well as sample processing workflows such as serum and plasma isolation, DNA extraction, RNA extraction, and cell line creation. In addition to STARLIMS, a software application called Crimson is leveraged at Brigham and Women’s Hospital to accession Biobank samples. Crimson also enables the Biobank to collect clinical discards for consented subjects. These are then shipped to our central storage facility and accessioned into STARLIMS. A sample indexing system called Enterprise Master Specimen Index (EMSI) enables the unique identification of samples regardless of their point of origin, and tracks the distribution of samples. Given the number of institutions within Partners HealthCare, some of the complexity of the Biobank infrastructure results from the requirements at each independent institution, and is being gradually simplified.

Although STARLIMS manages the laboratory processing of the specimens, an additional LIMS, Gateway for Integrated Genomic-Proteomic Applications and Data (GIGPAD), is used to manage workflows generating genomic data for both research and clinical operations. This genomic LIMS has been in operation for more than ten years, and provides functional support for different technologies including next generation sequencing, genotyping including Illumina iScan and Taqman, Sanger sequencing, and expression analysis services. The LIMS seamlessly integrates with the instruments and applications to help manage experiment batches and investigator requests. This system is also integrated with clinical genomics systems that provide reporting and translational capabilities. Currently, 25,000 Biobank specimens are being genotyped using the Illumina iScan platform and are being managed through GIGPAD. GIGPAD feeds data into bioinformatics pipelines that are responsible for data analysis and quality control. An in-house computing cluster of remote-desktop and compute nodes connected to very high speed storage is leveraged to enable this processing and to store the data. De-identified genomic data is then made available to investigators via their accounts on the cluster. While GIGPAD has aged fairly well, we are regularly looking at options to replace it for standard genotyping and sequencing workflows. Unfortunately, we have yet to find a tool that performs as well and is cost-effective.

Perhaps the most sophisticated component of our research IT infrastructure is the application that ties all these systems together for managing clinical phenotype data, specimen and recruitment data, genomic data, and for managing distribution of samples and data to the investigator community. A web-based tool called the Biobank Portal enables investigators to query the database of samples and data using any or all of the following criteria: clinical data, validated phenotypes, calculated controls, sample availability, and patient consent information. The Biobank Portal leverages the open source i2b2 software framework. In spring 2016, the Biobank Portal will be further enhanced to enable investigators to query the Biobank’s database based on genomic results. This investigator-facing portal is critical to the advancement of translational research at our medical institutions and greatly simplifies the mechanism through which samples and data are distributed to our investigators.

Most of the functional components in the Partners Biobank are integrated via a Service Oriented Architecture design, with Web Services providing data from one application to another as needed. This is a flexible approach used in modern enterprise infrastructures that allows applications to exchange data via reusable services [1]. Another type of integration, widely used in healthcare environments, is the exchange of HL7 messages [2]. This method is used in the Biobank infrastructure to comply with legacy systems. Also in use as an integration strategy in the Biobank infrastructure is the concept of shared databases, where two applications have access to the same database [3]. An example is the integration between the Consent Tracking system and the Enterprise Master Specimen Index, where the former is able to retrieve from the latter’s database the information of whether or not specimens exist for a specific subject.

## 3. Data Security and Integrity

A primary focus of the Biobank IT infrastructure is security and integrity of patient data. Data security is provided at several levels. The Biobank IT leverages the vast Partners information systems organization to keep its servers up to date with OS-level security patches and policies. Data is transferred using secure network connections between clients (browsers) and application servers and between application servers and the enterprise services. This transport level security is facilitated by Digital Certificates [4]. Users of the applications are given permissions to specific areas based on roles, and authentication is handled securely by the enterprise-wide LDAP. File system areas and databases are backed up on a regular basis to prevent data loss, and access to these is controlled via group-based authorization. In addition, data is stored in the online consent database in an encrypted state.

In addition to security, ensuring data integrity is a critical requirement of the IT infrastructure. Due to the complexity of our institution mentioned previously, the Biobank IT and operations make use of several practical strategies to ensure that data present in the Biobank and distributed to investigators is as accurate as possible. The strategies that were implemented to prevent manual error, include integration with clinical systems, double data entry [5], barcode [6] scanners and labels, and use of existing patient authentication infrastructure. An aim of all the systems requiring manual data entry was to limit the amount of information the Biobank staff was required to enter, and instead leverage data already recorded in clinical systems, as well as barcoded labels for specimens and paper based requisition forms. An example of which, applies to medical record numbers which allow the Biobank to associate the consented subject with phenotype data as well as auto-generated subject identifiers, without the staff member manually entering in all the associated data. Integration with clinical systems enables Biobank staff to confidently ascertain the identity of the patient at the point of consent by verifying such data as the patient’s name and date of birth. This integration with the clinical systems minimizes the potential of a paper based consent form being associated to the incorrect patient entry in electronic systems. In the case of electronic informed consent (eIC), which is completed online and does not require a lab staff to be present, the Biobank leverages existing patient authentication infrastructure to ensure unequivocal patient identification and achieve single sign-on [7] for patients.

In scenarios in which the systems are not integrated directly, the Biobank takes advantage of two distinct data entry applications, in which patient data is recorded; Constrack for consent data by research coordinators, and STARLIMS for collected specimens by lab staff. The consent and collected specimen databases are cross-checked every two weeks to ensure that there are no discrepancies between them. Any discrepancies identified through this cross check are immediately analyzed, resolved, and documented.

Downstream from the consent and collected specimen systems, the Biobank Portal provides a query-able view of the samples and genomic data, along with EHR data and derived clinical phenotype information in the Biobank to researchers interested in obtaining specimens. The data available in the Biobank Portal is transferred via a systems integration which keeps the consented patients and available specimens up to date. This dataset is also regularly compared with the consent and collected specimens data to ensure consistency and bring to light any issues to be addressed.

The Biobank’s consent and phenotype data provided by integration with clinical systems is supplemented with self-reported lifestyle and environmental data obtained from subjects via a survey administered either online or in paper form. Paper surveys are often completed by patients at home and mailed in to the Biobank. The staff enters this data in a web-based system that forces double data entry of each response by two different research coordinators. A reconciliation of the two entries is automatically done and a third research coordinator resolves any conflicts before the data is marked final.

## 4. Challenges of IT for Personalized Medicine

Building the IT infrastructure for a Biobank and a Translational Genomics Core presents some of the Information Technology challenges common in most industries: managing scope and preventing cost-overrun, focus on maintainability and supportability, decisions to purchase vendor-provided applications *vs.* in-house building of custom applications, ensuring usability and adequate performance of systems, and of foremost importance, security. An additional complexity at Partners HealthCare has been the complexity of the organization and the transitory nature of many of our clinical systems as the system transitions to Epic as its core clinical system.

The IT infrastructure of the Partners Biobank is a diverse ecosystem of applications, some pre-dating the Biobank and some that were built or purchased to fulfill specific functions. Where possible, the infrastructure was designed to leverage existing systems so as to make as little impact as possible on the workflows of staff at the associated hospitals, which necessarily interact with the Biobank especially in what concerns phlebotomy and specimen processing. Integrations with these systems were designed to be as lightweight and as flexible as possible and the Biobank IT team was able to define these integrations and lend technical support to other IT teams involved in implementing them.

A cost analysis is always performed when deciding to build a new system or purchase a vendor-provided system to fulfill a specific need. In the case of LIMS, for which mature products exist in the market, these were purchased and customized with participation of the IT team (STARLIMS). In three other cases—(1) the management of recruitment (Constrack) (2) the centralization of sample management across the several sites collecting and processing samples (EMSI) and (3) the management of mass communications with patients for the purpose of boosting recruitment—it was found that no available vendor application would provide the needed functionality. These systems were built in house and are actively maintained and enhanced based on ongoing use. This has definite advantages in the speed at which functionality can be added to accommodate business needs that arise. The IT team was able to quickly roll out an enhancement to the consent tracking system that allows paperless consent using mobile devices in the past year.

That being said, the Biobank IT team is constantly re-evaluating buy *vs.* build decisions, especially as the clinical IT infrastructure at Partners HealthCare is undergoing a massive change with the implementation of Epic. Epic, an enterprise Electronic Health Record software package, is replacing a number of vendor and homegrown systems that manage the electronic medical record, billing, patient scheduling, *etc.* It is possible that, once this implementation is complete, Epic may be able to simplify components of the Biobank infrastructure. For example, it is possible that Epic could replace Constrack to manage research consent tracking, but this remains to be seen. This constant evolution of both clinical systems and the ever changing landscape of Personalized Medicine, forces IT infrastructure to be robust, agile, and cost-effective. Three requirements will continue to be challenging for us to sustain; therefore, alternate solutions will continually be evaluated.
